# CD8+ T cells promote proliferation of benign prostatic hyperplasia epithelial cells under low androgen level via modulation of CCL5/STAT5/CCND1 signaling pathway

**DOI:** 10.1038/srep42893

**Published:** 2017-02-20

**Authors:** Yang Yang, Shuai Hu, Jie Liu, Yun Cui, Yu Fan, Tianjing Lv, Libo Liu, Jun Li, Qun He, Wenke Han, Wei Yu, Yin Sun, Jie Jin

**Affiliations:** 1 Department of Urology, Peking University First Hospital and Institute of Urology, Peking University, Beijing 100034, China; 2 National Research Center for Genitourinary Oncology, Beijing 100034, China; 3 Urogenital Diseases (male) Molecular Diagnosis and Treatment Center, Beijing 100034, China; 4 Department of Urology, Linyi People’s Hospital, Linyi 276003, Shandong, China; 5 Department of Radiation Oncology, University of Rochester Medical Center, Rochester 14642, NY, USA

## Abstract

Previous studies by our group have shown that low intra-prostatic dihydrotestosterone (DHT) induced BPH epithelial cells (BECs) to recruit CD8+ T cells. However, the influence of the recruited CD8+ T cells on BECs under a low androgen level is still unknown. Here, we found CD8+ T cells have the capacity to promote proliferation of BECs in low androgen condition. Mechanism dissection revealed that interaction between CD8+ T cells and BECs through secretion of CCL5 might promote the phosphorylation of STAT5 and a higher expression of CCND1 in BECs. Suppressed CCL5/STAT5 signals via CCL5 neutralizing antibody or STAT5 inhibitor Pimozide led to reverse CD8+ T cell-enhanced BECs proliferation. IHC analysis from Finasteride treated patients showed PCNA expression in BECs was highly correlated to the level of CD8+ T cell infiltration and the expression of CCL5. Consequently, our data indicated infiltrating CD8+ T cells could promote the proliferation of BECs in low androgen condition via modulation of CCL5/STAT5/CCND1 signaling. The increased secretion of CCL5 from the CD8+ T cells/BECs interaction might help BECs survive in a low DHT environment. Targeting these signals may provide a new potential therapeutic approach to better treat BPH patients who failed the therapy of 5α-reductase inhibitors.

Benign prostatic hyperplasia (BPH) is the most common urologic chronic and progressive disease in ageing men^1^. The incidence of BPH increases approximately 10% per decade of life after 50 years of age^2^,^3^. Despite the medical significance of BPH in ageing men, the pathogenesis of this disorder has not been completely elucidated. It is commonly believed that androgen/androgen receptor (AR) signaling plays key roles in the pathogenesis of BPH^4^. Finasteride, a 5α-reductase inhibitor, which suppresses testosterone conversion into dihydrotestosterone (DHT), has been one of the most commonly prescribed drugs for the management of BPH^5^. However, androgen/AR signaling pathway may not be the sole regulator of prostate growth as evidenced by the fact that over 25% of patients do not respond to 5α-reductase inhibitors (5ARIs)^6^,^7^,^8^.

It has been argued that BPH is an immune inflammatory disease and chronic inflammation is another key contributing factor to BPH^3^,^9^,^10^,^11^,^12^. A study of 282 BPH samples indicated that 81% of them stained positive for T cell markers (CD3), and patients with a higher inflammation level had larger prostate volumes and more severe symptoms^13^. Consistently, other studies also have shown that most chronic inflammatory cells in BPH tissues were T lymphocytes^14^,^15^. T lymphocytes infiltration in prostate tissues and the secretion of inflammatory cytokines within the prostatic gland are considered determinant factors in BPH pathogenesis and progression^12^,^16^.

Importantly, more recent reports have linked the androgen to inflammation, which might impact BPH progression. Studies from clinical samples and animal models suggested that androgen might play an anti-inflammatory effect in the prostate, while low androgen and high oestrogen levels might be associated with the infiltration of inflammatory cells in the prostate of BPH patients^17^,^18^,^19^,^20^,^21^,^22^, but the subset of T cells influenced by low intra-prostatic androgen still remained uncharacterized. Accordingly, our previous studies focused on the relationship between the intra-prostatic androgen level and T cells infiltration. We found that BPH patients treated with Finasteride 5 mg daily for longer than six months before surgery had more CD8+ T cells infiltration in the surrounding epithelial area in their prostatic tissue. We also demonstrated *in vitro* that a low androgen condition could induce BPH epithelial cells (BECs) to recruit CD8+ T cells via modulation of CCL5 secretion^23^. These findings supported the view that androgen plays an anti-inflammation effect in the prostate, and more specifically on the infiltration of CD8+ T cells. However, the consequences of infiltrated CD8+ T cells on prostatic epithelial cells in low androgen condition remain unclear.

In the present work, we focused on the effects of CD8+ T cells on the growth of BECs and demonstrated that infiltrated CD8+ T cells could promote the proliferation of BECs in the presence of low androgen. Mechanism dissection found that the infiltrated CD8+ T cells might go through modulation of CCL5/STAT5/CCND1 signaling to influence the growth of BECs.

## Results

### CD8+ T cells promoted the proliferation of BECs in the presence of low androgen

**Figure 1:**
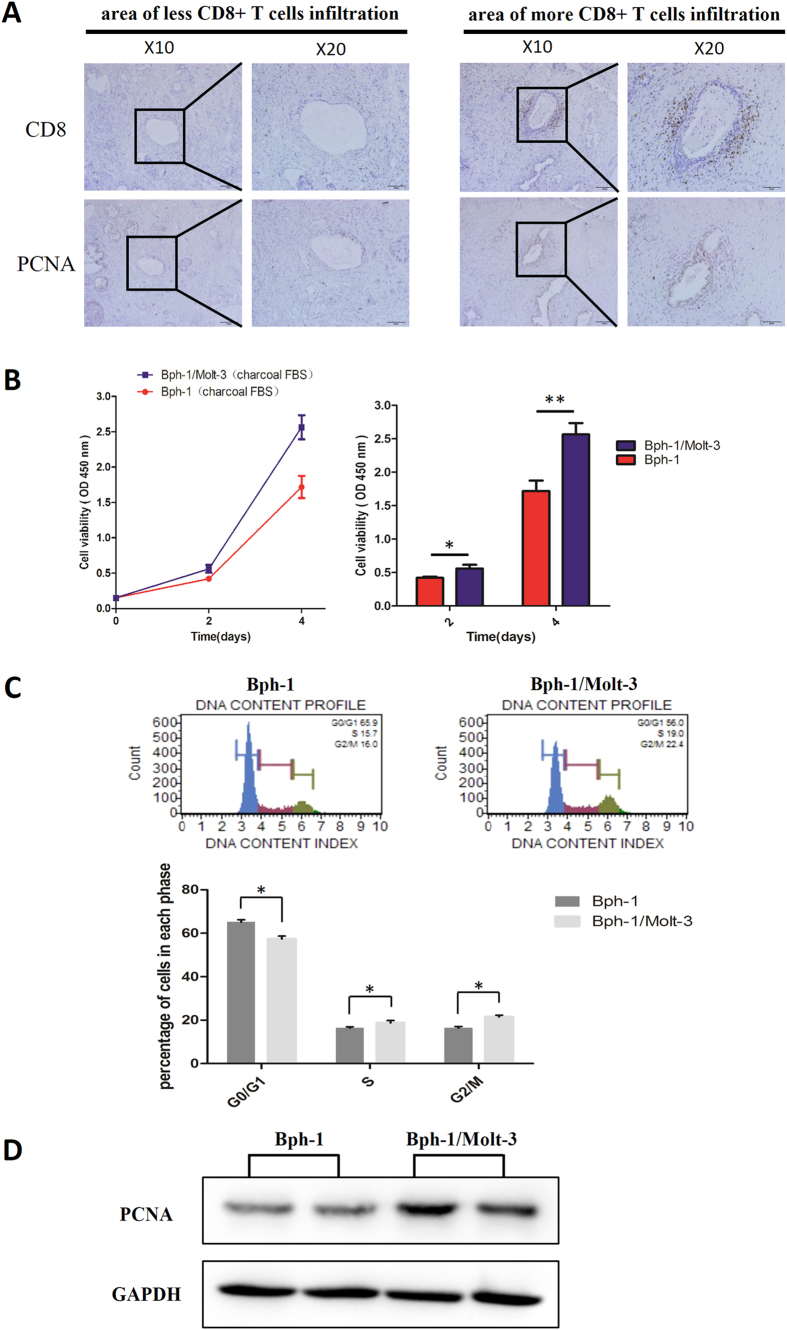
CD8+ T cells promoted the proliferation of BECs in the presence of low androgen. (**A**) IHC staining in the serial paraffin sections for CD8 and PCNA from BPH patients who treated with Finasteride at least six months. The left panel is an area with less CD8+ T cells infiltration, and the right panel is an area with more CD8+ T cells infiltration; scale bar: 100 *μ*m and 50 *μ*m. (**B**–**D**): Bph-1 cells were co-cultured with/without Molt-3 cells in low androgen condition for 4 days. (**B**) Bph-1 cells were detected with CCK8 at days 2 and 4. Data are shown as the average OD value of Bph-1 cells and are mean ± SD. *P < 0.05, **P < 0.01. (**C**) Bph-1 cells were harvested for cell cycle assay at days 4. Data are shown as the percentage of cells in each phase and are mean ± SD. *P < 0.05. (**D**) The proteins of Bph-1 cells were harvested at day 4. Western blot assay was performed using an antibody for PCNA. GAPDH was used as a loading control (full-length blots were presented in Supplementary Figure 1).

Early studies documented that one type of inflammatory cells, T-lymphocytes, can be attracted to the prostate tissue microenvironment and can promote the proliferation of prostatic epithelial cells^24^. Therefore, to investigate the influence of infiltrating CD8+ T cells on the growth of BECs in BPH samples with Finasteride treatment, we first examined the expression of CD8 and PCNA by IHC staining in serial paraffin sections. The results showed that CD8+ T cells were surrounding the epithelium area, and PCNA was mainly expressed in BECs. Moreover, we noticed that compared to the area of less CD8+ T cells infiltration, there was a higher PCNA expression in the BECs surrounded by more CD8+ T cells (Fig. 1A). Separately, we used the CCK8 assay to examine the cell growth of Bph-1 cells during co-culture with Molt-3 cells (CD8+ T-lymphocytic cell line)^24^,^25^, and we found that Molt-3 cells could significantly promote the cell growth of Bph-1 cells in the low androgen condition, at both day 2 and 4 (Fig. 1B). Consistently, compared to Bph-1 cells cultured alone in a low androgen condition, Bph-1 cells showed a significant increase in cells with the S phase and G2/M phase DNA content with a corresponding decrease in the number of cells with G0/G1 DNA content after Bph-1/Molt-3 cells co-culture in the low androgen condition group (Fig. 1C). The increased cell proliferation of Bph-1 during co-culturing with Molt-3 cells can also be confirmed by the increased expression of PCNA by the western blot analysis (Fig. 1D, the uncropped images were presented in Supplementary Figure 1).

Taken together, these results from Fig. 1 suggest that more infiltrating CD8+ T cells migrating towards the glandular epithelium area of the prostate in BPH patients could promote the proliferation of BECs in the condition of low androgen.

### CD8+ T cells promoted the proliferation of BECs via increased secretion of CCL5 in low androgen level

**Figure 2:**
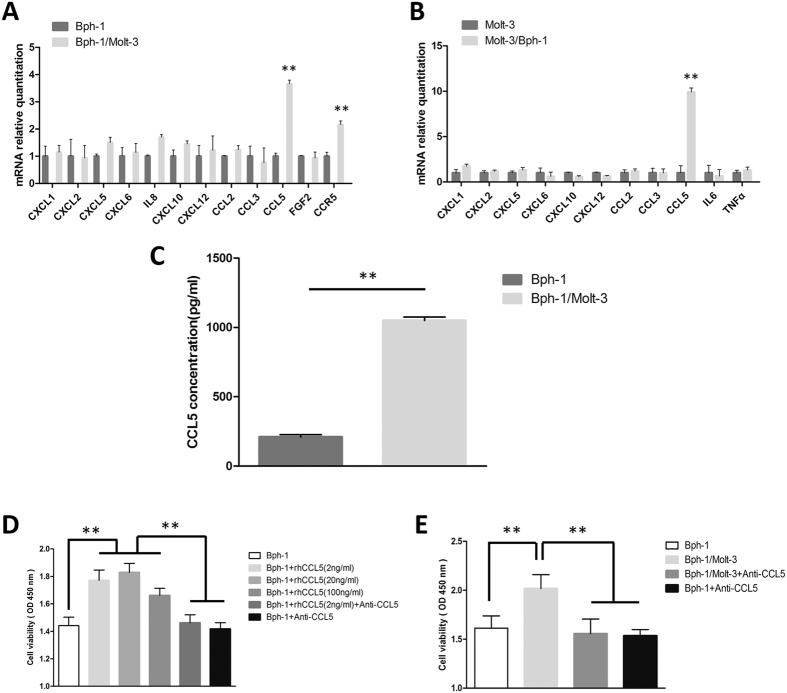
CD8+ T cells promoted the proliferation of BECs via increased secretion of CCL5 in low androgen level. (**A**) Q-PCR analysis of cytokine/chemokine expression in Bph-1 cells at days 2. In the co-culture group, CCL5 and CCR5 mRNA were up-regulated in Bph-1 cells compared to the mono-culture group. **P < 0.05. (**B**) Q-PCR analysis of cytokine/chemokine expression levels in Molt-3 cells at days 2. In the co-culture group, CCL5 mRNA were up-regulated in Molt-3 cells compared to the mono-culture group. **P < 0.05. (**C**) ELISA analysis of CCL5 in the conditioned media isolated from the co-culture group or the mono-culture group at days 4. The concentration of CCL5 in co-culture group was higher than mono-culture group. **P < 0.05. (**D**) CCK8 assay showed addition of rhCCL5 at 2, 20, 100 ng/ml increased the proliferation of Bph-1 cells at days 4 compared to control group, addition of rhCCL5 (2 ng/ml) with neutralizing CCL5 antibody (2 *μ*g/ml) group, or addition of neutralizing CCL5 antibody (2 *μ*g/ml) group, respectively. **P < 0.05. (**E**) CCK8 assay showed neutralizing CCL5 antibody (2 *μ*g/ml) reversed the Molt-3 cells-enhanced Bph-1 cells proliferation effect at days 4. **P < 0.05.

Emerging evidence indicates that many growth factors and chemokines through the autocrine or paracrine actions of prostatic epithelial cells, stromal cells and inflammatory cells, induce the proliferation of BECs in various circumstances^24^,^26^,^27^,^28^,^29^,^30^,^31^,^32^,^33^. To identify which inflammatory cytokines/chemokines are responsible for CD8+ T cell-induced BECs proliferation in the low androgen condition, we performed quantitative PCR analysis of the selected inflammatory cytokines/chemokines that were potentially involved in this cross-talk between CD8+ T cells and BECs in the above co-culture system. Of these cytokine transcripts, we found a remarkable increase of CCL5 expression both in Bph-1 cells and Molt-3 cells after co-culture in the low androgen condition (Fig. 2A and B). Interestingly, the mRNA levels of CCR5 were also up-regulated in Bph-1 cells from the co-culture group (Fig. 2A). Furthermore, ELISA assay also confirmed the increased induction of CCL5 at the protein level in the conditioned media of Bph-1/Molt-3 co-culture with a low androgen condition (Fig. 2C).

It is established that CCL5 induces the proliferation, migration, and invasiveness of prostate cells^30^,^34^. Similarly, to investigate the CCL5-induced proliferation effect of BECs, we seeded Bph-1 cells in 96-well plates (1 × 10^3^ cells/well) and found that rhCCL5 (2, 20, 100 ng/ml in low androgen media) could significantly increase Bph-1 cells proliferation in low androgen media for 4 days by CCK8 assay (Fig. 2D). Significantly, the anti-CCL5 neutralizing antibody could negate this increased cell proliferation in the co-culture condition, confirming the role of CCL5 in mediating the stimulation of cell growth (Fig. 2E).

Together, the results from Fig. 2 show that the increased secretion of CCL5 from the CD8+ T cells/BECs interaction plays a crucial role in the CD8+ T cell-induced BECs proliferation in the condition of low androgen.

### CD8, CCL5, PCNA expression in clinical samples with Finasteride treatment

**Figure 3:**
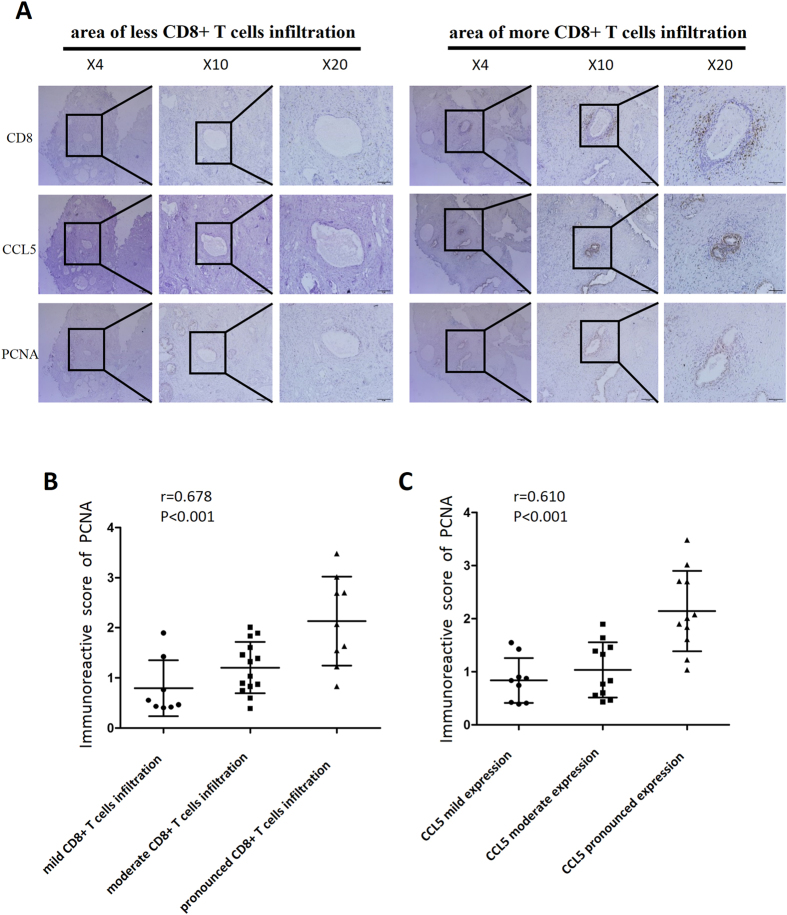
CD8, CCL5, PCNA expression in clinical samples with Finasteride treatment. (**A**) IHC staining for CD8, CCL5, PCNA in serial paraffin sections of tissue specimens from 31 BPH patients treated with Finasteride at least six months; scale bar: 200 *μ*m, 100 *μ*m and 50 *μ*m. Compared to the area of less CD8+ T cells infiltration (left panel), the CCL5 and PCNA expression showed higher in the BPH epithelial cells where surrounded by more CD8+ T cells infiltration (right panel). (**B**) Spearman rank correlation showed there was a positive correlation between the degree of CD8+ T cells infiltration and the expression of PCNA (r = 0.678, *P* < 0.001). (**C**) The expression intensity of CCL5 and the expression of PCNA were also positively correlated by analysis of Spearman rank correlation (r = 0.610, *P* < 0.001).

To further demonstrate the role of CCL5 in the CD8+ T cells promotion of BECs proliferation under low androgen condition, we examined the expression of CD8, CCL5 and PCNA with IHC staining in serial paraffin sections of BPH tissues from 31 patients who were treated with Finasteride for at least six months. The results revealed that the PCNA and CCL5 expression were higher in the BECs surrounded by more CD8+ T cells infiltration than in the area with less CD8+ T cells infiltration (Fig. 3A). The Spearman rank correlation analysis indicated that there was a positive correlation between the degree of CD8+ T cell infiltration and the expression of PCNA (r = 0.678, P < 0.001) (Fig. 3B), while the staining intensity of CCL5 and the expression of PCNA were also positively correlated (r = 0.610, P < 0.001) (Fig. 3C).

### Mechanism dissection of how increased CCL5 promoted the proliferation of BECs in low androgen level

**Figure 4:**
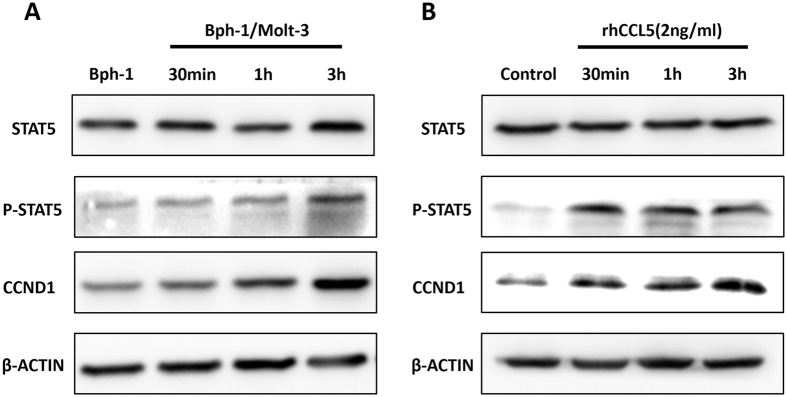
Mechanism dissection of how increased CCL5 promoted the proliferation of BECs in low androgen level. (**A**) Bph-1 cells were treated with conditioned media from the co-culture group for 30 min, 1 h and 3 h compared to the mono-culture group. Western blot assay was performed using antibodies specific for total STAT5, Phospho-STAT5 and CCND1. (**B**) Bph-1 cells were treated with rhCCL5 (2 ng/ml) at the indicated times. Western blot assay was performed using antibodies specific for total STAT5, Phospho-STAT5 and CCND1. β-ACTIN was used as a loading control (full-length blots were presented in Supplementary Figure 2).

Early reports suggested that CCL5 could rapidly induce JAK-STAT5 phosphorylation^35^,^36^. It has also been reported that STAT5 phosphorylation regulated the expression of CCND1 in prostate cancer cells^37^,^38^. To determine whether CCL5/JAK-STAT5/CCND1 played a role in promoting BEC cell proliferation in the condition of low androgen condition, we compared their expression in the Bph-1 cells that were cultured in conditioned media from Bph-1/Molt-3 cells co-culture or that from Bph-1 cells mono-culture. As shown in Fig. 4A, in the presence of co-culture media that resulted in the activation of STAT5 as shown with the up-regulation of STAT5 phosphorylation followed by higher expression of CCND1 in Bph-1 cells. The results also revealed that there was a time-dependent up-regulation of STAT5 phosphorylation and the expression of CCND1 by prolonging the treatment of the co-culture media (the uncropped images were presented in Supplementary Figure 2).

Next, to determine whether CCL5 was sufficient in promoting BECs proliferation under a low androgen condition, Bph-1 cells were cultured with 2 ng/ml of rhCCL5 in low androgen media for the indicated times and analysed for the expression of STAT5, Phospho-STAT5 and CCND1 by western blot assay. Similar to the effect of conditioned media from the co-culture group, we found that STAT5 phosphorylation was up-regulated in Bph-1 cells upon treatment with rhCCL5 at 30 min, 1 h and 3 h compared with the control group. Consistently, the expression of CCND1 showed a time-dependent up-regulation by prolonging the treatment of rhCCL5 (Fig. 4B, the uncropped images were presented in Supplementary Figure 2).

These results suggest that increased CCL5 produced from the CD8+ T cells/BECs interaction might activate the STAT5/CCND1 signaling pathway, which likely promoted the proliferation of BECs in the low androgen condition.

### The STAT5 inhibitor Pimozide reversed CCL5/STAT5/CCND1 signaling pathway and CD8+ T cell-enhanced BECs proliferation

**Figure 5:**
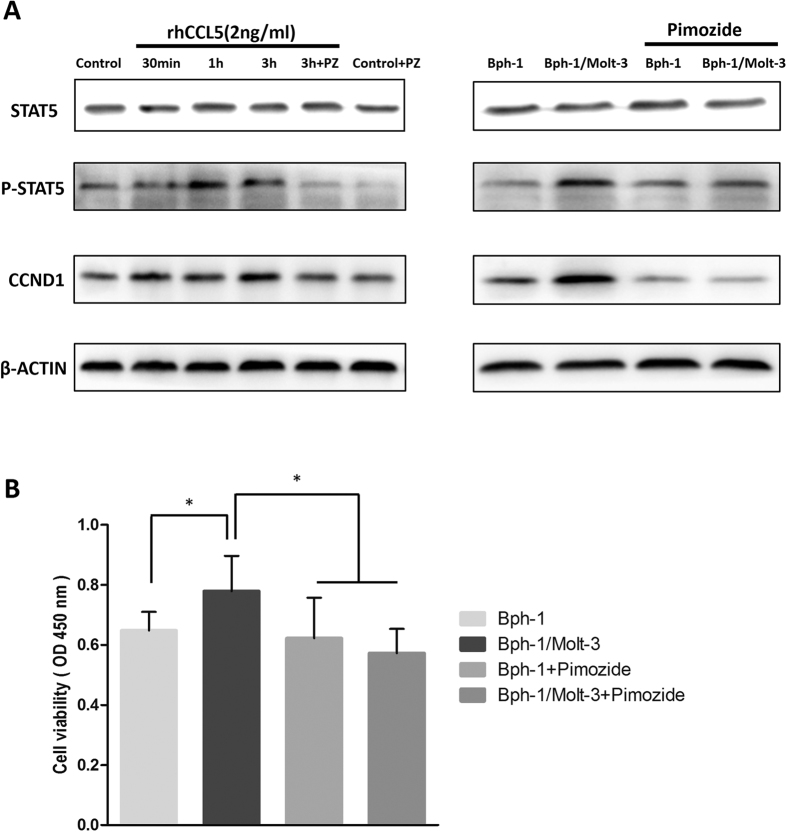
The STAT5 inhibitor Pimozide reversed CCL5/STAT5/CCND1 signaling pathway and CD8+ T cell-enhanced BECs proliferation. (**A**) Western blot results showed the up-regulation of Phospho-STAT5 and CCND1 were reversed by Pimozide (PZ). Before the treatment of rhCCL5 or conditioned media (3 h), Bph-1 cells were treated with the Pimozide (10 *μ*M) for 2 hours. β-ACTIN was used as a loading control (full-length blots were presented in Supplementary Figure 3). (**B**) CCK8 assay showed that the addition of Pimozide (10 *μ*M) reversed the Molt-3 cells-enhanced Bph-1 cells proliferation at days 2. *P < 0.05.

To further confirm the functional contribution of STAT5 in this regulation, the STAT5 inhibitor, Pimozide, which specifically suppresses the phosphorylation of STAT5^39^, was used to inhibit STAT5 function in Bph-1 cells during the treatment of rhCCL5 or conditioned media from Bph-1/Molt-3 cells co-culture. We examined Pimozide (10 *μ*M) on the treatment of 2 ng/ml of rhCCL5 or conditioned media as described above, followed by the analysis of the expression of STAT5, phospho-STAT5 and CCND1 by western blot assay. The results showed that Pimozide reversed the up-regulated expression of STAT5 phosphorylation and CCND1 in Bph-1 cells after the treatment with rhCCL5 or conditioned media (Fig. 5A, the uncropped images were presented in Supplementary Figure 3).

Next, we applied the interruption assay via adding Pimozide for 2 hours in Bph-1 cells before a 2-day treatment of conditioned media. After that, the CCK8 assay was used to detect the proliferation of Bph-1 cells, and we found that blocking the activation of STAT5 in Bph-1 cells partially reversed the Molt-3 cells-enhanced Bph-1 cells proliferation (Fig. 5B).

Together, the results from Figs 1, 2, 3, 4, 5 suggest that the signaling axis of CCL5/STAT5/CCND1 in BEC cells could be a key step to mediate the influence of infiltrated CD8+ T cells on the proliferation of BECs.

## Discussion

To date, there has been no consensus on the etiology of BPH. Theories of BPH pathogenesis, including altered oestrogen/androgen balance^40^,^41^,^42^, increased oxidative stress^43^,^44^, metabolic syndromes^45^,^46^, chronic inflammation^3^, epithelial-mesenchymal transition^47^, and altered activity of autonomic nerves^48^, have been postulated. Consistent with the role of androgen in promoting human prostatic epithelial cells growth^49^, Finasteride, an inhibitor of androgen synthesis, can alleviate BPH symptoms through the significant prostatic epithelial cells atrophy and apoptosis thus reduction of prostate volume^50^,^51^,^52^,^53^ and relieve LUTS secondary to BPH^54^,^55^. However, at least 25–30% of patients still do not respond to this therapy^6^,^56^. For the first time, our study identified that infiltrated CD8+ T cells could promote the survival of prostatic epithelial cells even in the condition of low androgen. These findings suggested that increased CD8+ T cells infiltration toward the glandular epithelium area of the prostate may be one of the possible reasons that a subset of BPH patients did not respond to the anti-androgen therapy and still need surgical treatment.

These findings are consistent with the increasing consensus that the major cause of BPH progression is prostatic inflammation. In addition, it is reported that T lymphocytes consist of 70–80% prostatic immune inflammatory cells^3^,^14^,^57^. For this reason, many studies have focused on discussing the relationships between T cells and the progression of prostate diseases. Kathleen *et al*. found that CD4+ T cells and CD8+ T cells increased the risk of clinical progression of BPH^58^. In an autopsy study, Alexandre *et al*. showed that men who had chronic inflammation, mostly T cells, were 6.8 times more likely to have a higher BPH score than individuals with no chronic inflammation^59^. Other studies also indicated that increased infiltration of CD4+ T cells and CD8+ T cells within the tumor was associated with a poor outcome in prostate cancer patients^15^. Furthermore, cytokines such as IL-6, IL-8, IFN-r, FGF-2, TGF-β, CCL2, CXCL10 and CXCL12, produced by T cells and senescent BPH cells via autocrine/paracrine secretion, are involved in altering tissue remodeling and hyperplastic growth at each stage of BPH^11^,^12^,^26^,^28^,^29^,^31^,^33^,^47^. As a hormone-dependent disease, BPH was studied with a focus on the relationships between androgen levels and immune inflammation in the prostate. There is a viewpoint that androgen may play an anti-inflammatory effect in the prostate^17^,^20^,^21^. Indeed, we showed that BPH patients treated with Finasteride could have more CD8+ T cells infiltration surrounding the epithelial area, suggesting that androgen plays an important role in the regulation of immune homeostasis, and a low androgen may lead to an inflammation disorder in a part of the prostate in BPH patients. Then, the present study found that the increased infiltration of CD8+ T cells, which may directly promote prostatic epithelial growth after infiltration into prostate tissues via interaction with prostatic epithelial cells in a low androgen level. This is in agreement with previous studies showing inflammatory cells could be attracted to the prostate tissue microenvironment and could promote the proliferation of prostatic epithelial cells^24^.

Early studies indicated that chemokine family members might play important roles in the proliferation of BECs^27^,^28^,^29^,^30^,^31^,^33^. Here, we applied Q-PCR to examine the cytokines/chemokines expression in the CD8+ T cell line – Molt-3 cells and the BPH epithelial cell line – Bph-1 cells, and the ELISA assay to identify the secretion of CCL5 in conditioned media at a low androgen level. We found that chemokine CCL5 secretion, which plays a critical role in the enhanced BECs proliferation, was increased during the interaction between CD8+ T cells and BECs with a reduction of androgen. We also found that the CCL5 co-receptor CCR5 was up-regulated in Bph-1 cells after being co-cultured with Molt-3 cells in the low androgen condition^60^. Significantly, CCL5 expression was also confirmed in the clinical samples we collected. These results suggested that CCL5 may be the key factor in CD8+ T cell-enhanced BECs proliferation in low androgen condition, substituting for androgen/AR signaling to make BECs survive in a reduced androgen level.

To further dissect the downstream signals of CCL5 on the growth of BECs in the condition of low androgen, we applied western blot assay to examine the expression of STAT5 which has been implicated in the growth and progression of many malignancies, including haematopoietic, prostate, and breast cancer^61^,^62^,^63^. In prostatic diseases, STAT5 has been identified and validated as a potential therapeutic target protein in prostate cancer^64^,^65^. Here, for the first time, we found that Molt-3 cells might affect BECs through CCL5 activating STAT5 by up-regulation of STAT5 phosphorylation followed by a higher expression of CCND1 in BECs. Intriguingly, interruption approaches using the STAT5 inhibitors, such as Pimozide, could reverse both STAT5 phosphorylation and the up-regulation of CCND1, and suppress the Molt-3 cells-enhanced BECs proliferation. These findings suggest that the STAT5 inhibitors might not only be a potential therapy for prostate cancer but also for BPH patients who failed anti-androgen therapy.

**Figure 6:**
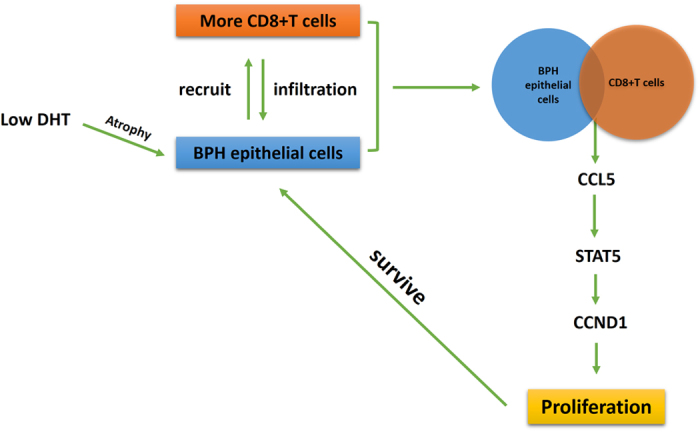
Low intra-prostatic DHT promotes CD8+ T cells infiltration in BPH prostate tissue. Then, increased secretion of CCL5 from CD8+ T cells/BECs interaction could promote the proliferation of BPH epithelial cells in the condition of low androgen via activation of the STAT5/CCND1 signaling pathway.

In conclusion, the most striking finding of the present study was that infiltrating CD8+ T cells could promote the proliferation of BECs in a low androgen condition via the modulation of the CCL5/STAT5/CCND1 signaling pathway (Fig. 6). The increased secretion of CCL5 originates from the CD8+ T cells/BECs interaction, which might help BECs survive in the condition of low DHT, thus illustrating a possible cause for treatment failure of 5ARIs. Anti-inflammation therapies via targeting the CCL5 or STAT5 signaling pathway in patients with BPH may be warranted in the future.

## Materials and Methods

### Patients

The patients were selected based on medication from the electronic medical record system of 921 patients with BPH who underwent transurethral resection of the prostate (TURP) to relieve lower urinary tract symptoms (LUTS) between January 2007 and December 2011 in the Department of Urology, Peking University First Hospital, Beijing, China. Patients who had urinary tract infection, or prostatitis, or previous prostate related surgery or the history of urethral catheterization were excluded from this study. Prostate tissues were obtained from 31 BPH patients treated with Finasteride 5 mg daily for at least six months before surgery. The prostate specimens were examined microscopically by two pathologists to determine a diagnosis of BPH without prostate cancer or prostatic intraepithelial neoplasia.

### Cell Lines, Co-culture Experiments

The BPH epithelial cell line, Bph-1, was purchased from Keygen Biotech Co., Ltd (KG1008, NJ, China) and the CD8+ T-lymphocytic cell line, Molt-3 cells^24^,^25^, was purchased from the American Type Culture Collection (CRL-1552, Rockville, MD, USA). Cell lines were grown in RPMI-1640 media (SH30809.01B, HyClone, South Logan UT, USA) containing 1% penicillin G and 1% streptomycin, supplemented with 10% fetal bovine serum (FBS). All cell lines were cultured in a standard 37 °C humidified incubator with 5% carbon dioxide. Charcoal-stripped FBS and phenol-red-free media were used as a low androgen environment for culturing cells.

*In vitro*, 6-well transwell plates (0.4 *μ*m pore size, polycarbonate membrane inserts) were used for the co-culture experiments (3460/3450, Corning, NY, USA). Bph-1 cells (2 × 10^4^ cells/well) with/without Molt-3 cells (2 × 10^4^ cells/well) in low androgen media were treated with 10% charcoal FBS (SH30068.03, HyClone, South Logan UT, USA). After Bph-1/Molt-3 cells co-culture (co-culture group) or Bph-1 cells cultured alone (mono-culture group) for 4 days, the conditioned media, Bph-1 cells and Molt-3 cells were harvested for the subsequent experiments.

### Reagents and Antibodies

Antibodies used for immunohistochemistry and western blot included rabbit anti-CD8(+) (RM-9116-S1, Thermo Fisher Scientific, Cheshire, UK), rabbit anti-CCL5 (ab9679, Abcam, Cambridge, UK), mouse anti-PCNA (2586, Cell Signaling Technology, MA, USA), rabbit anti-GAPDH (2118, Cell Signaling Technology, MA, USA), mouse anti-β-ACTIN (3700, Cell Signaling Technology, MA, USA), rabbit anti-STAT5 (9363, Cell Signaling Technology, MA, USA), rabbit anti-phospho-STAT5 (9314, Cell Signaling Technology, MA, USA) and rabbit anti-CCND1 (2978, Cell Signaling Technology, MA, USA). The reagents used in the *in vitro* experiment included recombinant human CCL5 (rhCCL5), anti-CCL5 neutralizing antibody and Pimozide were purchased from R&D systems (278-RN, MAB678, 0937 Minneapolis, MN, USA). rhCCL5 was reconstituted in phosphate buffered saline (PBS) and adjusted to a final concentration of 2, 20, 100 ng/ml. For blocking the proliferation effect of rhCCL5 and conditioned media, the anti-CCL5 neutralizing antibody was reconstituted in PBS and adjusted to a final concentration of 2 *μ*g/ml. The STAT5 inhibitor Pimozide was used for interruption assay at a final concentration of 10 *μ*M (dissolved in DMSO). All reagents were used in a low androgen condition to evaluate the proliferation effect and the mechanism dissection of CD8+ T cells or rhCCL5 on BECs.

### Cell Proliferation Assay

Bph-1 cells at 5 × 10^3^ cells/well were plated into the lower chamber of the 24-well transwell plates and cultured in RPMI-1640 with a low androgen level overnight. Then, Molt-3 cells at 5 × 10^3^ cells/well were plated onto the upper chamber with 0.4 *μ*m pore polycarbonate membrane inserts in the co-culture group, or only had fresh media added into the upper chamber in the mono-culture group. During a 4-day culture, the Bph-1 cells growth status was detected at days 2 and 4 by the Cell Counting Kit-8 (CCK8) according to the manufacturer’s protocol (CK04, Dojindo, Japan). Briefly, the Bph-1 cells were incubated in serum-free medium with a CCK8 solution (1:10) for 2 h in the 37 °C humidified incubator. The absorbance value was measured using a Varioskan Flash (Thermo Scientific) at 450 nm. Furthermore, in the low androgen condition, the CCK8 assay was also used to detect the proliferation of Bph-1 cells plated into 96-well plates (1 × 10^3^ cells/well) after treatment with conditioned media, rhCCL5, anti-CCL5 neutralizing antibody, or Pimozide.

### Cell Cycle Assay

For the cell cycle analysis, after a 4-day cell culture in low androgen condition, Bph-1 cells in the co-culture group and the mono-culture group were harvested and immobilized in 75% ethyl alcohol at 4 °C overnight. Then, Bph-1 cells were washed twice with PBS. Finally, the washed cells were resuspended in 0.5 ml PBS with propidium iodide staining solution (50 *μ*g/ml propidium iodide, 0.2% Triton X-100, 50 *μ*g/ml RNase A). After a 30-minute incubation protected from light, the cells were analysed by the Muse Cell Analyzer (0500-3115, Millipore, MA, USA). The cell cycle distribution was determined using the Muse 1.4 Analysis software.

### Western blot Assay

Bph-1 cells were washed twice in PBS and lysed with RIPA buffer containing 1% protease inhibitors, 0.5% phosphatase inhibitors and 1 mM PMSF (KGP250, Keygen Biotech, NJ, China). The protein concentration in the cell lystate solution was determined by BCA protein assay (NCI3227CH, Thermo, Pittsburgh, PA, USA). The cell lystate was mixed with 5X SDS-PAGE loading buffer (Amresco). Equivalent protein quantities were heated at 95 °C for 10 min before separation on precasted 10–15% SDS polyacrylamide gels (Bio-Rad). The proteins were electro-transferred onto nitrocellulose membranes that were blocked in Tris-buffered saline plus 0.05% Tween-20 (TBS-T) containing 5% non-fat dried milk for 1 h. Then, the membranes were incubated with primary antibodies overnight at 4 °C in TBS-T containing 5% bovine serum albumin (BSA). After washing in TBS-T buffer, the membranes were incubated with goat anti-horseradish peroxidase-conjugated secondary antibody (1:1000; Invitrogen) for 1 h at room temperature in TBS-T. The membranes were then washed with TBS-T buffer, and the signals were visualized using a Western-blot chemiluminescent reagent according to the manufacturer’s protocol (P90719, Millipore, MA, USA). The following primary antibodies were used: rabbit anti-GAPDH (1:1000, Cell Signaling Technology), mouse anti-β-ACTIN (1:1000, Cell Signaling Technology), mouse anti-PCNA (1:1000, Cell Signaling Technology), rabbit anti-STAT5 (1:1000, Cell Signaling Technology), rabbit anti- Phospho-STAT5 (1:1000, Cell Signaling Technology) and rabbit anti-CCND1 (1:1000, Cell Signaling Technology).

### Quantitative PCR

Total RNA was extracted from Bph-1 cells and Molt-3 cells using TRIzol (15596-018, Invitrogen, Grand Island, NY, USA). According to the manufacturer’s protocol, cDNA was synthesized from 1 *μ*g RNA, using a high capacity cDNA reverse transcription kit (4368813, Applied Biosystems, CA, USA). Quantitative real-time PCR (qRT-PCR) was conducted using the GoTaq qPCR Master Mix (A6001, Promega, Madison, WI, USA) for a two-step cycling protocol with an Applied Biosystems 7500 Fast Real-Time PCR system to determine the level of mRNA expression of target genes. The relative expression levels were calculated using the 2−ΔΔCt method. The quantities of all targets from the test samples were normalized to the GAPDH housekeeping gene.

### ELISA Assay

Conditioned media collected from Bph-1 cells cultured alone or Bph-1/Molt-3 cells co-culture at 4 days were also used for the detection of CCL5 by human CCL5 Quantikine ELISA Kit (DRN00B, R&D Systems, Minneapolis, MN, USA) according to the manufacturer’s protocol.

### Immunohistochemistry

The expression of CD8, CCL5 and PCNA were analysed by serial paraffin sections immunohistochemistry (IHC) to study the relationship among CD8+ T cells, CCL5 and proliferation status of prostatic epithelial cells. The prostate tissues from TURP surgery were fixed in 4% buffered formalin overnight at 4 °C and then dehydrated in an ascending ethanol series, routinely embedded in paraffin, and sectioned at 5 *μ*m. After conventional deparaffinization, hydration, and antigen retrieval, endogenous peroxidase was inactivated by 3% hydrogen peroxide. The primary antibodies of the rabbit anti-CD8 (+) (1: 50, Thermo), rabbit anti-CCL5 (2 *μ*g/ml, Abcam) and mouse anti-PCNA (1: 4000, Cell Signaling Technology) were used for incubation at 4 °C overnight. The primary antibody was recognized by the biotinylated secondary antibody (PK-4001, Vector Labs, Burlingame, CA, USA) at room temperature for 30 min and visualized by the VECTASTAIN ABC peroxidase system and peroxidase substrate DAB kit (SK-4100, Vector Labs).

The amount of CD8+ T cells infiltration (grade:1–3), proteins expression of CCL5 (grade: 1–3) and the average immunoreactive score of PCNA (IOD) were analysed using the Image-Pro Plus 6.0 software (Media Cybernetics, Rockville, MD, USA). The degree of prostatic CD8+ T cells infiltrated was based on the mean number of CD8+ T cells aggregates in the prostate stroma under 200 x field (1, mild infiltration: <400 cells/mm^2^; 2, moderate infiltration: >400 cells/mm^2^, <800 cells/mm^2^; 3, pronounced infiltration: >800 cells/mm^2^). The expression intensity of CCL5 was assessed semiquantitatively, and a 3-tiered system (1 weak, 2 moderate, 3 strong) was used. All pathologic assessments were performed by two experienced pathologists in a blinded fashion.

### Statistical Analysis

The data are expressed as the mean ± SD of at least 3 independent experiments. Statistical analyses between the two groups were analysed by paired t-test. IHC data were analysed through Spearman rank correlation test. P values < 0.05 was considered statistically significant. All statistical analyses were performed using the SPSS 17.0 (SPSS Inc., Chicago, IL).

### Ethical standards

All procedures performed in the studies involving human participants were in accordance with the ethical standards of the research committee of Peking University First Hospital and the principles of the Declaration of Helsinki. All participants provided written informed consent before taking part in the study. All experimental protocol were approved by the research committee of Peking University First Hospital.

## Additional Information

**How to cite this article**: Yang, Y. *et al*. CD8+T cells promote proliferation of benign prostatic hyperplasia epithelial cells under low androgen level via modulation of CCL5/STAT5/CCND1 signaling pathway. *Sci. Rep.* **7**, 42893; doi: 10.1038/srep42893 (2017).

**Publisher's note:** Springer Nature remains neutral with regard to jurisdictional claims in published maps and institutional affiliations.

## Supplementary Material

Supplementary Figures
