# Supplementary material for: CD8+ T cells promote proliferation of benign prostatic hyperplasia epithelial cells under low androgen level via modulation of CCL5/STAT5/CCND1 signaling pathway
Source: Sci Rep. 2017 Feb 20;7:42893. doi: 10.1038/srep42893 (PMC5316951; doi:10.1038/srep42893)

CD8+T cells promote proliferation of benign prostatic hyperplasia epithelial cells under low androgen level via modulation of CCL5/STAT5/CCND1 signaling pathway

*Yang Yang, Shuai Hu, Jie Liu, Yun Cui, Yu Fan, Tianjing Lv, Libo Liu, Jun Li, Qun He, Wenke Han, Wei Yu, Yin Sun, and Jie Jin**

Supplementary Figure 1

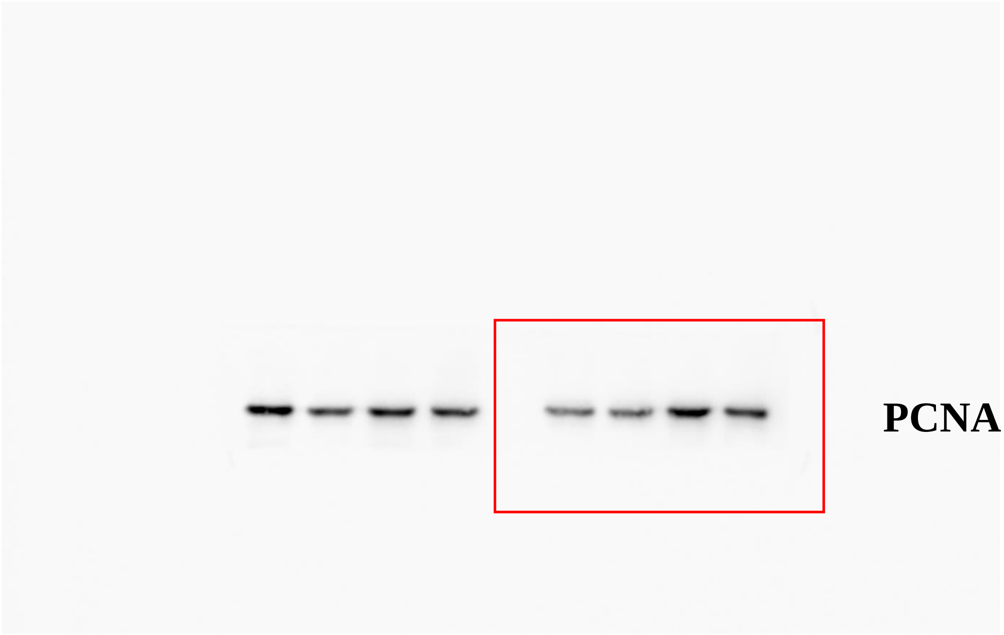

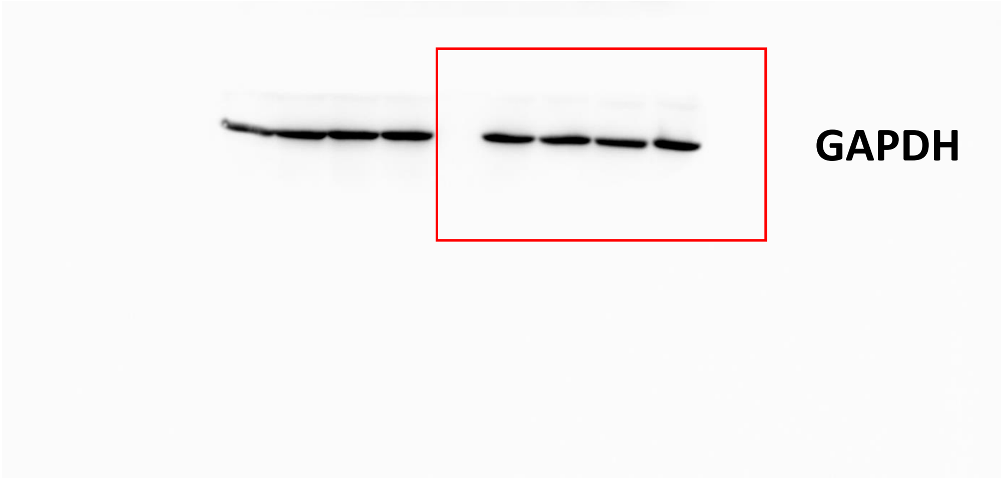

Supplementary Figure 2

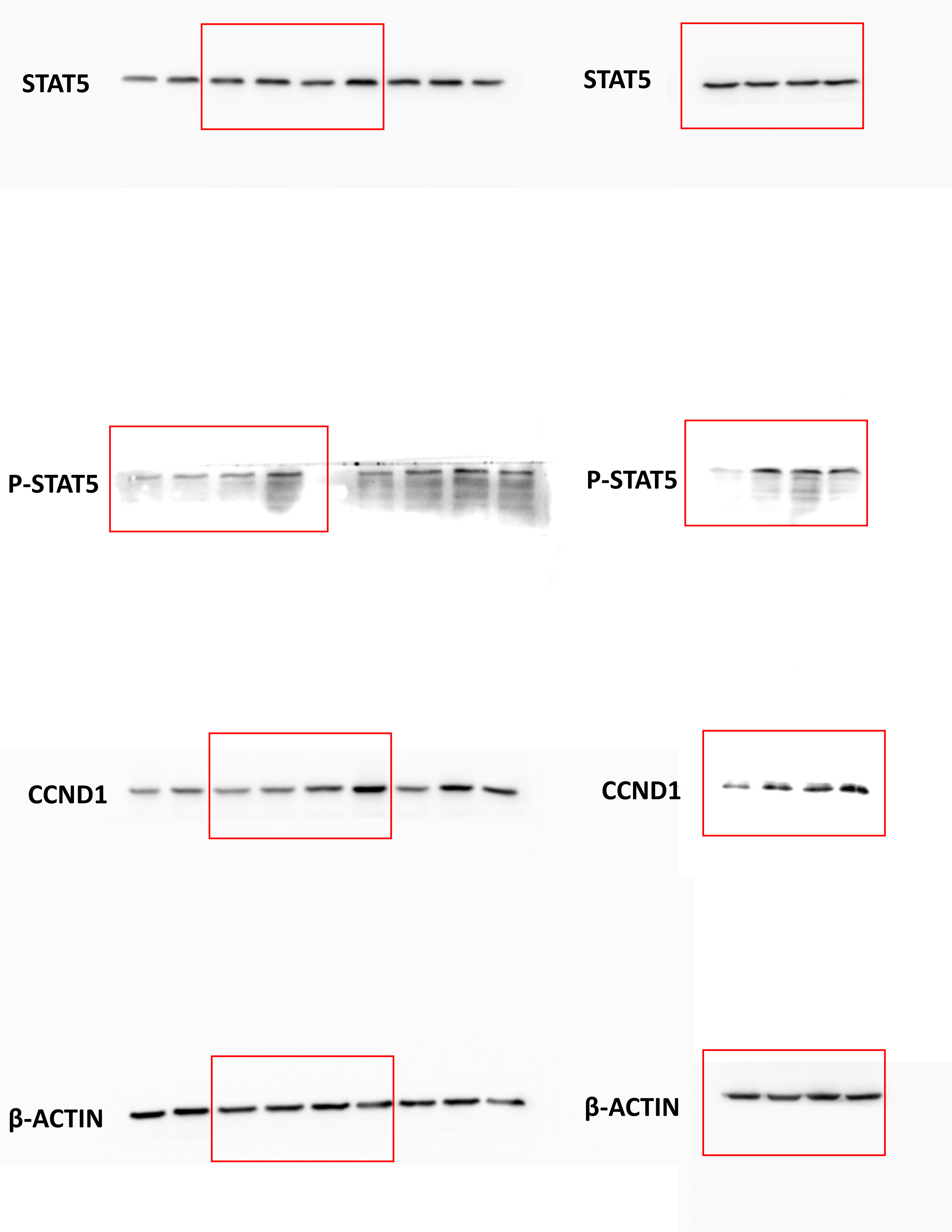

Supplementary Figure 3

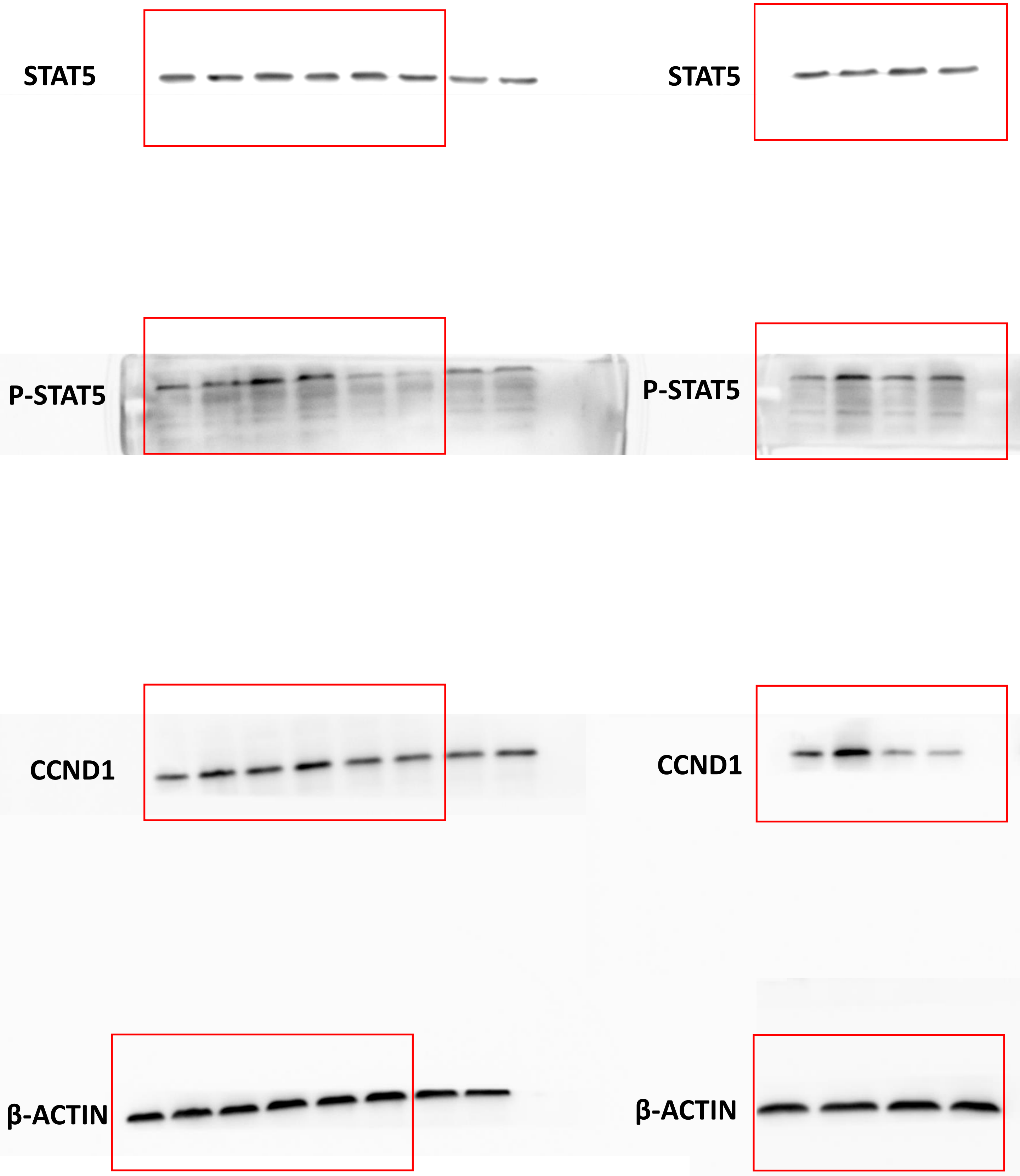

Supplement: Supplementary Figures [file srep42893-s1.pdf]
